# Distribution and susceptibility of *ERCC1/XPF* gene polymorphisms in Han and Uygur women with breast cancer in Xinjiang, China

**DOI:** 10.1002/cam4.3547

**Published:** 2020-10-16

**Authors:** Hongtao Li, Linghui Zhou, Jing Ma, Yuyao Zhu, Jingjing Fan, Na Li, Yi Zheng, Tong Sha, Zhen Zhai, Binlin Ma, Zhijun Dai

**Affiliations:** ^1^ Department of Breast Head and Neck Surgery The 3rd Affiliated Teaching Hospital of Xinjiang Medical University (Affiliated Tumor Hospital Urumqi China; ^2^ Department of Breast Surgery The First Affiliated Hospital College of Medicine Zhejiang University Hangzhou China; ^3^ Department of Oncology The Second Affiliated Hospital of Xi’an Jiaotong University Xi’an China

**Keywords:** breast cancer, gene polymorphisms, Han, susceptibility, Uygur

## Abstract

This study aimed to explore the roles of *ERCC1*/*XPF* gene polymorphisms in the occurrence of breast cancer in the Uygur and Han ethnic groups in Xinjiang, China. Single nucleotide polymorphisms (SNPs) were detected by TaqMan real‐time PCR. The rs11615 G>A and rs2276466 C>G variant frequencies were higher in Uygur patients with breast cancer than in Han patients, while the frequency of rs2298881 C>A was higher in Han patients. We found that rs2298881 C>A (CA vs. CC: OR = 0.35, 95% CI = 0.20‐0.60; AA vs. CC: OR = 0.13, 95% CI = 0.04‐0.34; CA + AA vs. CC: OR = 0.33, 95% CI = 0.18‐0.51; AA vs. CA + CC: OR = 0.24, 95% CI = 0.08‐0.62; CA vs. AA + CC: OR = 0.49, 95% CI = 0.29‐0.82) was associated with a reduced breast cancer risk and rs3212986 C>A (AA vs. CC: OR = 4.80, 95% CI = 1.79‐15.29,; CA+AA vs. CC: OR = 1.71, 95% CI = 1.06‐2.77; AA vs. CA+CC: OR = 4.12, 95% CI =1.58‐12.89) and rs11615 G > A (AA vs. GG: OR = 3.49, 95% CI =1.54‐8.55; GA + AA vs. GG: OR = 1.98, 95% CI = 1.21‐3.27; AA vs. GA+GG: OR = 2.87, 95% CI = 1.30‐6.85) were associated with an elevated breast cancer risk among Uygur individuals. In addition, Uygur patients with breast cancer with 2‐3 combined risk genotypes of *ERCC1* had a higher risk than patients with 0‐1 risk genotypes (OR = 2.91; 95% CI = 1.54‐5.71, *p* = 0.001). However, we failed to detect a statistically significant association between *ERCC1*/*XPF* polymorphisms and breast cancer risk in five genetic models among Han individuals. Our results showed that *ERCC1*/*XPF* gene polymorphisms predispose Uygur individuals to breast cancer; this finding should be verified by further large‐scale analyses.

## Graphical abstract

Distribution of ERCC1/XPF gene polymorphisms in Han and Uygur women with breast cancer was different. ERCC1/XPF gene polymorphisms predispose Uygur individuals to breast cancer.

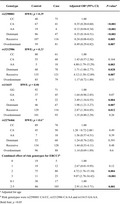

## 1 INTRODUCTION

Breast cancer is one of the most serious cancers threatening the health of women worldwide. According to the World Cancer Statistics, in 2018, approximately 2,100,000 women were diagnosed with breast cancer, accounting for 24.2% of all cancers, ranking first, and approximately 630,000 people died of breast cancer worldwide, accounting for 15% of the total cancer‐related deaths, also ranking first.^1^ In 2015, there were about 268,600 new cases of breast cancer in women and 69,500 deaths in China.^2^ Compared with countries in Europe and the Americas, the incidence of breast cancer in China is relatively low. However, over the past 20 to 30 years, the incidence of breast cancer in China has increased at twice the average rate worldwide, and the mortality rate is also increasing.^3^ The detrimental effects of breast cancer on the health of women have become a serious public health issue in China. Although existing treatments have greatly improved prognosis, some patients with breast cancer still have poor outcomes.

Individual genetic factors may play an important role in breast cancer susceptibility, treatment responses, and prognosis.^4^ To date, genome‐wide association studies (GWAS) and multiple large‐scale repeated sequencing studies have identified more than 70 single nucleotide polymorphisms (SNPs) related to breast cancer, including the high‐penetrance breast cancer‐related genes *BRCA1* (breast cancer associated gene 1) and *BRCA2* (Breast cancer associated gene 2), moderate‐penetrance genes *CHEK2* (checkpoint kinase 2) and *BRIP1* (BRCA1 interacting protein C‐terminal helicase 1), and low‐penetrance genes *FGFR2* (fibroblast growth factor receptor 2), *TNRC9* (also known as TOX3, TOX high mobility group box family member 3), *MAP3K1* (mitogen‐activated protein kinase kinase kinase 1), and *LSP1* (lymphocyte specific protein 1).^5^, ^6^ However, these susceptible genetic variants account for only a small proportion of variation in breast cancer risk; moreover, correction for multiple testing in GWAS can eliminate potential SNPs.^7^ Therefore, more gene polymorphisms associated with susceptibility to breast cancer need to be identified. The nucleotide excision repair pathway eliminates twisted helix DNA damage in a multi‐step "shear and repair" reaction, and defects in the pathway may lead to cancer.^8^ Some previous studies indicate that SNPs in the nucleotide excision repair pathway are associated with susceptibility to certain cancers.^9^, ^10^

Excision repair cross‐complementation group 1 (*ERCC1*) and *XPF* (also known as ERCC4, excision repair cross‐complementation group 4) encode two proteins involved in the nucleotide excision repair pathway. Owing to the important role of the ERCC1/XPF complex in the DNA repair process, exploring the role of *ERCC1*/*XPF* gene polymorphisms in cancer risk has been a major focus of research.^11^

In Xinjiang, China, the incidence of breast cancer is second only to cervical cancer. Han and Uygur are two major ethnic groups in Xinjiang, accounting for 90% of the total population. Although there is no definite epidemiological information about the incidence of breast cancer among Han and Uygur populations in Xinjiang, it is obviously lower in the Uygur population than in the Han population. According to the dynamic changes in the number of hospitalized individuals over the past 5 years, the number of patients with breast cancer of Uygur ethnicity has increased, with an average annual growth rate of 2.11%, while patients of Han ethnicity have fluctuated, with an average annual growth rate of −11.44%. Another study has shown that the incidence of breast cancer in Xinjiang Uygur women is low; however, the age of onset is relatively early (i.e., 36‐50 years), most patients are stage II and III, and the prognosis is poor.^12^ Therefore, it is important to explore differences in risk factors for breast cancer between Xinjiang Uygur and Han populations. The purpose of our study was to explore the associations between *ERCC1*/*XPF* polymorphisms and breast cancer risk and to compare their distributions in Uygurs and Hans to improve our understanding of their roles in the pathogenesis of breast cancer in different races.

## 2 MATERIALS AND METHODS

### 2.1 Ethics statement

Prior to the study, all participants provided written informed consent. The study was approved by the Ethics Committee of the Third Affiliated Hospital of Xinjiang Medical University.

### 2.2 Study population

A total of 140 Uygur patients with breast cancer, 141 Uygur healthy controls, 265 Han patients with breast cancer, and 374 Han healthy controls were included in the study. All patients were women and were consecutively recruited between December 2017 and December 2018 at the Third Affiliated Hospital of Xinjiang Medical University. All patients were diagnosed by pathological biopsy in the hospital and did not undergo radiotherapy or chemotherapy before surgery. All patients receive treatment at the time of sample collection. All individuals in the control groups were healthy females who underwent a physical examination at the same hospital during the same time period. Clinical information for patients was obtained from hospital medical records, including name, age, race, menopausal status, tumor volume, TNM stage, estrogen receptor (ER) status, progesterone receptor (PR) status, human epidermal growth factor receptor‐2 (HER2) status, ki67 (also known as MKI67, marker of proliferation ki67) status, and P53 (also known as protein 53 or tumor protein 53) status. Information for individuals in the control group was obtained from the medical examination center system, including name, race, and age.

### 2.3 Genotyping assay

After the patients and healthy controls signed the informed consent form, we collected 5 ml of the subjects’ peripheral blood into an EDTA‐anticoagulation test tube. The dbSNP database (http://www.ncbi.nlm.nih.gov/) was used to select potential functional SNPs in *ERCC1*/*XPF*.^13^, ^14^ A kit provided by Beijing Kangwei Century Biology Company (Beijing, China) was used to extract DNA from whole blood. SNP genotyping was performed by TaqMan real‐time PCR. SNP primers were designed and synthesized by Applied Biosystems (Foster City). The probes for variant and wild‐type allele were labeled with fluorescent dyes VIC and FAM, respectively. PCR reaction was performed with a 384‐well plate (each well with a reaction volume of 5 μl). The PCR machine identified the genotypes based on the relative fluorescence intensity of VIC and FAM.^15^, ^16^ Four negative controls and eight duplicate samples were set in each 384‐well plate for quality control. Finally, four SNPs (rs2298881, rs3212986, and rs11615 in *ERCC1* and rs2276466 in *XPF*) were successfully genotyped.

### 2.4 Statistical analysis

Hardy–Weinberg equilibrium (HWE) in the control population was evaluated. Six inheritance models were used to assess cancer susceptibility. The chi‐squared test was used to assess differences in genotype and allele frequencies. Logistic regression, adjusting for age, was used to calculate the association between SNPs and breast cancer susceptibility. The GTEx (genotype‐tissue expression, https://www.gtexportal.org/) portal was used to assess the biological effects of rs2298881 C>A and rs11615 G>A on *ERCC1* gene expression.^17^ All statistical tests were two‐sided, and statistical significance was evaluated at the 0.05 α‐level. All results were calculated using R (version 3.5.1).

## 3 RESULTS

### 3.1 Distribution of *ERCC1*/*XPF* polymorphisms in distinct ethnic groups

As determined by a chi‐squared test, the distributions of *ERCC1* rs2298881 C>A (*p* < 0.001), *ERCC1* rs11615 G>A (*p* < 0.001), and *XPF* rs2276466 C>G (*p* = 0.002) differed significantly between Uygur and Han patients with breast cancer. Similar results were found for the two alleles. The detailed results are shown in Table 1.

**TABLE 1:** Distribution difference of *ERCC1*/*XPF* polymorphism between Uygur and Han nationality

Gene	SNP	Allele		race	Genotype frequency			χ^2^	*p* value	Allele frequency		*χ* ^2^	*p* value
		A	B		AA	AB	BB			A	B		
*ERCC1*	rs3212986	C	A	Han	109(41.9)	132(50.8)	19(7.3)	4.28	0.118	350(67.3)	170(32.7)	0.28	0.599
				Uygurs	61(43.9)	60(43.2)	18(12.9)			182(65.5)	96(34.5)		
*ERCC1*	rs11615	G	A	Han	196(75.4)	47(18.1)	17(6.5)	29.36	<0.001	439(84.4)	81(15.6)	29.83	<0.001
				Uygurs	71(51.4)	45(32.6)	22(15.9)			187(67.8)	89(32.2)		
*ERCC1*	rs2298881	C	A	Han	110(42.1)	116(44.4)	35(13.4)	14.41	<0.001	336(64.4)	186(35.6)	14.99	<0.001
				Uygurs	75(61.5)	41(33.6)	6(4.9)			191(78.3)	53(21.7)		
*XPF*	rs2276466	C	G	Han	195(73.6)	61(23.0)	9(3.4)	12.51	0.002	451(85.1)	79(14.9)	13.17	<0.001
				Uygurs	78(56.5)	50(36.2)	10(7.2)			206(74.6)	70(25.4)		

Bold font: *p* < 0.05.

### 3.2 Associations between *ERCC1*/*XPF* polymorphisms and breast cancer susceptibility

We found significant associations between four SNPs and breast cancer susceptibility in the allelic genetic models among the Han and Uygur groups; the details are shown in Table 2. However, we failed to detect a statistically significant association between the four SNPs and breast cancer risk in the other five genetic models for the Han ethnicity (Table 3). As shown in Table 4, significant associations was observed between rs2298881 C>A (CA vs. CC: OR = 0.35, 95% CI = 0.20‐0.60, *p* < 0.001; AA vs. CC: OR = 0.13, 95% CI = 0.04‐0.34, *p* < 0.001; CA+AA vs. CC: OR = 0.33, 95% CI = 0.18‐0.51, *p* < 0.001; AA vs. CA+CC: OR = 0.24, 95% CI = 0.08‐0.62, *p* = 0.005; CA vs. AA+CC: OR = 0.49, 95% CI = 0.29‐0.82, *p* = 0.007), rs3212986 (AA vs. CC: OR = 4.80, 95% CI = 1.79‐15.29, *p* = 0.003; CA+AA vs. CC: OR = 1.71, 95% CI = 1.06‐2.77, *p* = 0.028; AA vs. CA+CC: OR = 4.12, 95% CI = 1.58‐12.89, *p* = 0.007), rs11615 (AA vs. GG: OR = 3.49, 95% CI = 1.54‐8.55, *p* = 0.004; GA+AA vs. GG: OR = 1.98, 95% CI = 1.21‐3.27, *p* = 0.007; AA vs. GA+GG: OR = 2.87, 95% CI = 1.30‐6.85, *p* = 0.012) and breast cancer susceptibility in the Uygur population. In addition, we found that Uygur patients with breast cancer with 2‐3 combined risk genotypes of *ERCC1* had a higher risk than that of individuals with 0‐1 risk genotypes (OR = 2.91; 95% CI = 1.54‐5.71, *p* = 0.001).

**TABLE 2:** Allelic genetic models among the Han and Uygur nationalities

Gene	SNP	Allele		Case		Control		OR (95% CI)	*p*
		A	B	A	B	A	B		
Han									
*ERCC1*	rs2298881	C	A	336	309	448	280	1.47(1.19‐1.82)	<0.001
*ERCC1*	rs3212986	C	A	350	353	496	224	2.23(1.80‐2.77)	<0.001
*ERCC1*	rs11615	G	A	439	555	620	106	7.39(5.81‐9.41)	<0.001
*XPF*	rs2276466	C	G	451	521	616	128	5.56(4.42‐6.99)	<0.001
Uygurs									
*ERCC1*	rs2298881	C	A	191	86	147	105	0.63(0.44‐0.90)	0.011
*ERCC1*	rs3212986	C	A	182	178	215	65	3.24(2.29‐4.57)	<0.001
*ERCC1*	rs11615	G	A	187	206	221	55	4.43(3.10‐6.32)	<0.001
*XPF*	rs2276466	C	G	206	188	223	59	3.45(2.43‐4.89)	<0.001

Bold font: *p* < 0.05.

**TABLE 3:** Logistic regression analysis for the correlation of *ERCC1* and *XPF* polymorphisms with Han breast cancer risk

Genotype	Control	Case	Adjusted OR^a^ (95% CI)	*p*‐value^a^
rs2298881 HWE: *p* = 0.85				
CC	137	110	1.00	
CA	174	116	0.83 (0.58‐1.18)	0.292
AA	53	35	0.79 (0.46‐1.33)	0.38
Dominant	227	151	0.82 (0.59‐1.15)	0.247
Recessive	311	226	0.89 (0.55‐1.42)	0.626
Overdominant	190	145	0.88 (0.63‐1.22)	0.428
rs3212986 HWE: *p* = 0.97				
CC	167	109	1.00	
CA	162	132	1.27 (0.90‐1.78)	0.178
AA	31	19	0.99 (0.52‐1.85)	0.97
Dominant	193	151	1.22 (0.88‐1.71)	0.231
Recessive	329	241	0.89 (0.47‐1.62)	0.697
Overdominant	198	128	1.26 (0.91‐1.76)	0.16
rs11615 HWE: *p* = 0.11				
GG	269	196	1.00	
GA	82	47	0.84 (0.55‐1.27)	0.42
AA	12	17	1.88 (0.87‐4.16)	0.11
Dominant	94	64	0.99 (0.67‐1.43)	0.94
Recessive	351	243	1.94 (0.91‐4.29)	0.09
Overdominant	281	213	0.81 (0.54‐1.22)	0.32
rs2276466 HWE: *p* = 0.93				
CC	256	195	1.00	
CA	104	61	0.71 (0.48‐1.03)	0.0719
AA	12	9	0.96 (0.38‐2.36)	0.93
Dominant	116	70	0.73 (0.51‐1.05)	0.0899
Recessive	360	256	1.05 (0.41‐2.57)	0.92
Overdominant	268	204	0.71 (0.48‐1.03)	0.072
Combined effect of risk genotypes for ERCC1^b^				
0	46	32	1.00	
1	59	37	0.92 (0.49‐1.72)	0.79
2	197	158	1.20 (0.72‐2.01)	0.48
3	38	24	1.02 (0.50‐2.05)	0.96
0‐1	105	69	1.00	
2‐3	235	182	1.23 (0.85‐1.78)	0.28

Bold font: *p* < 0.05.

^a^ Adjusted for age.

^b^ Risk genotypes were rs2298881 CA/CC, rs3212986 CA/AA, and rs11615 GA/AA.

**TABLE 4:** Logistic regression analysis for the correlation of *ERCC1* and *XPF* polymorphisms with Uygur breast cancer risk

Genotype	Control	Case	Adjusted OR^a^ (95% CI)	*p*‐value^a^
rs2298881 HWE: *p* = 0.29				
CC	40	75	1.00	
CA	67	41	0.35 (0.20‐0.60)	<0.001
AA	19	6	0.13 (0.04‐0.34)	<0.001
Dominant	86	47	0.33 (0.18‐0.51)	<0.001
Recessive	107	116	0.24 (0.08‐0.62)	0.005
Overdominant	59	81	0.49 (0.29‐0.82)	0.007
rs3212986 HWE: *p* = 0.23				
CC	80	61	1.00	
CA	55	60	1.43 (0.57‐2.36)	0.164
AA	5	18	4.80 (1.79‐15.29)	0.003
Dominant	60	78	1.71 (1.06‐2.77)	0.028
Recessive	135	121	4.12 (1.58‐12.89)	0.007
Overdominant	85	79	1.17 (0.72‐1.89)	0.53
rs11615 HWE: *p* = 0.06				
GG	92	71	1.00	
GA	37	45	1.64 (0.96‐2.85)	0.07
AA	9	22	3.49 (1.54‐8.55)	0.004
Dominant	46	67	1.98 (1.21‐3.27)	0.007
Recessive	129	116	2.87 (1.30‐6.85)	0.012
Overdominant	101	93	1.35 (0.80‐2.29)	0.26
rs2276466 HWE: *p* = 0.67				
CC	89	78	1.00	
CA	45	50	1.20（0.72‐2.00）	0.49
AA	7	10	1.56 (0.57‐4.51)	0.39
Dominant	52	60	1.24 (0.76‐2.02)	0.391
Recessive	134	128	1.44 (0.53‐4.11)	0.48
Overdominant	96	88	1.14 (0.69‐1.89)	0.6
Combined effect of risk genotypes for ERCC1^b^				
0	19	5	1.00	
1	19	12	2.67 (0.81‐9.95)	0.12
2	75	80	4.72 (1.76‐15.10)	0.004
3	11	23	9.07 (2.78‐34.42)	<0.001
0‐1	38	17	1.00	
2‐3	86	103	2.91 (1.54‐5.71)	0.001

Bold font: *p* < 0.05.

^a^ Adjusted for age.

^b^ Risk genotypes were rs2298881 CA/CC, rs3212986 CA/AA, and rs11615 GA/AA.

### 3.3 Stratification Analysis

To further explore the association between *ERCC1*/*XPF* polymorphisms and breast cancer susceptibility, we performed a stratified analysis according to age, TNM stage, ER status, PR status, HER2 status, Ki67 status, and P53 status. As shown in Table 5, among the Han population, *ERCC1* rs2298881 C>A was associated with a reduced risk of breast cancer in individuals ≥50 years old or with positive expression of P53. *XPF* rs2276466 C>G was also associated with a lower risk of breast cancer in patients aged <50 years, stage I+II, with positive expression of ER, positive expression of PR, or negative expression of Ki67. Similar associations for different P53 expression states were found. In the Uygur population, rs2298881 C>A was associated with a reduced risk of breast cancer with positive expression of HER2 or p53, irrespective of age, TNM stage, ER, PR, and P53 expression status. Rs3212986 C>A was related to negative expression of PR, HER2, or Ki67. Rs11615 G>A was related to the risk of breast cancer in patients <50 years of age, with negative expression of ER, positive expression of PR, or positive expression of p53. A similar association was found for patients with breast cancer with different stages and Ki67 statuses; the details are shown in Table 6.

**TABLE 5:** Stratification analysis for the association between *ERCC1*/*XPF* gene genotypes and Han breast cancer susceptibility.

Variable	rs2298881case/control		OR (95% CI)	*p*	rs3212986case/control		OR (95% CI)	*p*	rs11615case/control		OR (95% CI)	*p*	rs2276466case/control		OR (95% CI)	*p*
	CC	CA+AA			CC	CA+AA			GG	GA+AA			CC	CG+GG		
**Han**																
Age																
<50	47/94	83/137	1.21(0.78‐1.90)	0.4	58/105	71/121	1.06(0.69‐1.64)	0.79	100/167	32/60	0.89(0.54‐1.46)	0.65	108/166	25/69	0.56(0.33‐0.92)	0.03
≥50	63/43	68/90	0.52(0.31‐0.85)	0.001	51/62	80/72	1.35(0.83‐2.21)	0.23	96/102	32/34	1.00(0.57‐1.75)	1	87/90	45/47	0.99(0.60‐1.64)	0.97
Stage																
0 + I+II	77/137	107/227	0.81(0.56‐1.18)	0.27	80/167	104/193	1.16(0.80‐1.67)	0.43	137/269	47/94	1.04(0.68‐1.57)	0.87	143/256	44/116	0.62(0.41‐0.94)	0.03
III+IV	33/137	42/227	0.72(0.43‐1.21)	0.21	29/167	45/193	1.42(0.85‐2.40)	0.19	57/269	17/94	0.91(0.49‐1.63)	0.76	50/256	26/116	1.09(0.63‐1.83)	0.76
ER																
Negative	29/137	48/227	0.89(0.53‐1.52)	0.68	33/167	42/193	1.14(0.68‐1.92)	0.62	59/269	18/94	0.96(0.52‐1.72)	0.9	46/256	32/116	1.41(0.84‐2.36)	0.19
Positive	81/137	101/227	0.74(0.51‐1.07)	0.11	76/167	107/193	1.27(0.88‐1.83)	0.21	135/269	46/94	1.02(0.67‐1.54)	0.92	147/256	38/116	0.54(0.35‐0.82)	0.005
PR																
Negative	47/137	70/227	0.82(0.53‐1.29)	0.39	53/167	63/193	1.08(0.70‐1.67)	0.73	89/269	30/94	1.04(0.63‐1.68)	0.89	76/256	44/116	1.18(0.75‐1.83)	0.47
Positive	63/137	79/227	0.74(0.50‐1.10)	0.14	56/167	86/193	1.37(0.92‐2.05)	0.13	105/269	34/94	0.98(0.61‐1.53)	0.92	117/256	26/116	0.47(0.28‐0.75)	0.002
HER2																
Negative	62/137	85/227	0.80(0.53‐1.19)	0.27	65/167	82/193	1.11(0.75‐1.66)	0.59	109/269	40/94	1.14(0.73‐1.76)	0.57	110/256	39/116	0.73(0.47‐1.13)	0.16
Positive	43/137	64/227	0.85(0.54‐1.33)	0.46	44/167	62/193	1.29(0.83‐2.03)	0.26	81/269	23/94	0.85(0.49‐1.42)	0.55	80/256	29/116	0.75(0.46‐1.21)	0.25
Ki67																
Negative	25/137	24/227	0.55(0.30‐1.00)	0.051	25/167	24/193	0.87(0.47‐1.58)	0.64	31/269	19/94	1.81(0.96‐3.36)	0.06	44/256	6/116	0.29(0.11‐0.65)	0.006
Positive	85/137	125/227	0.88(0.61‐1.26)	0.48	84/167	125/193	1.32(0.92‐1.89)	0.13	163/269	45/94	0.85(0.55‐1.28)	0.43	149/256	64/116	0.88(0.60‐1.28)	0.5
P53																
Negative	22/137	22/227	0.57(0.30‐1.08)	0.08	21/167	22/193	0.94(0.50‐1.78)	0.84	27/269	17/94	1.86(0.95‐3.55)	0.06	38/256	6/116	0.33(0.12‐0.76)	0.016
Positive	19/137	16/227	0.47(0.23‐0.96)	0.04	15/167	21/193	1.27(0.63‐2.59)	0.51	24/269	11/94	1.38(0.63‐2.90)	0.4	33/256	3/116	0.19(0.04‐0.54)	0.007

Bold font: *p* < 0.05. ER: estrogen receptor.

HER2, human epidermal growth factor receptor‐2; PR, progesterone receptor.

**TABLE 6:** Stratification analysis for the association between *ERCC1*/*XPF* gene genotypes and Uygur breast cancer susceptibility

Variable	rs2298881case/control		OR (95% CI)	*p*	rs3212986case/control		OR (95% CI)	*p*	rs11615case/control		OR (95% CI)	*p*	rs2276466case/control		OR (95% CI)	*p*
	CC	CA+AA			CC	CA+AA			GG	GA+AA			CC	CG+GG		
**Uygurs**																
Age																
<50	42/27	29/62	0.30(0.15‐0.57)	<0.001	37/58	46/42	1.72(0.96‐3.10)	0.07	36/66	47/32	2.69(1.48‐4.98)	0.001	50/63	33/38	1.09(0.60‐1.99)	0.77
≥50	33/16	18/24	0.30(0.12‐0.71)	0.007	24/22	32/18	1.63(0.72‐3.72)	0.24	35/26	20/14	1.06(0.45‐2.51)	0.89	28/26	27/14	1.79(0.78‐4.21)	0.71
Stage																
0 + I+II	48/40	32/86	0.32(0.18‐0.58)	<0.001	41/80	52/60	1.67(0.98‐2.86)	0.06	48/92	44/46	1.91(1.11‐3.32)	0.02	52/89	42/52	1.33(0.77‐2.27)	0.30
III+IV	27/40	15/86	0.25(0.12‐0.53)	0.004	20/80	26/60	1.88(0.94‐3.79)	0.07	23/92	23/46	2.08(1.04‐4.20)	0.04	26/89	18/52	1.06(0.51‐2.14)	0.88
ER																
Negative	39/40	17/86	0.20(0.10‐0.41)	<0.001	28/80	37/60	1.64(0.89‐3.06)	0.12	31/92	34/46	2.27(1.21‐4.28)	0.01	40/89	24/52	1.00(0.52‐1.87)	0.99
Positive	36/40	30/86	0.39(0.21‐0.72)	0.003	33/80	41/60	1.70(0.96‐3.03)	0.07	40/92	33/46	1.68(0.94‐3.03)	0.08	38/89	26/52	1.59(0.89‐2.85)	0.12
PR																
Negative	27/40	15/86	0.29(0.13‐0.63)	0.002	17/80	31/60	2.38(1.17‐4.99)	0.018	29/92	18/46	1.28(0.61‐2.65)	0.51	28/89	19/52	1.18(0.57‐2.41)	0.66
Positive	48/40	32/86	0.31(0.17‐0.55)	<0.001	44/80	47/60	1.44(0.85‐2.47)	0.18	42/92	49/46	2.35(1.37‐4.08)	0.002	50/89	41/52	1.39(0.80‐2.41)	0.24
HER2																
Negative	16/40	15/86	0.46(0.21‐1.04)	0.06	12/80	23/60	2.44(1.13‐5.51)	0.025	25/92	11/46	0.89(0.38‐1.95)	0.77	19/89	17/52	1.71(0.80‐3.67)	0.17
Positive	27/40	19/86	0.40(0.18‐0.84)	0.02	21/80	30/60	2.01(1.00‐1.14)	0.05	30/92	20/46	1.60(0.76‐3.34)	0.21	26/89	23/52	1.29(0.62‐2.64)	0.49
Ki67																
Negative	13/40	14/86	0.50(0.21‐1.17)	0.11	16/80	13/60	1.08(0.48‐2.42)	0.85	12/92	17/46	2.84(1.26‐6.60)	0.013	15/89	14/52	1.61(0.71‐3.63)	0.25
Positive	62/40	33/86	0.25(0.14‐0.45)	<0.001	45/80	65/60	1.98(1.19‐3.34)	0.01	59/92	50/46	1.84(1.09‐3.15)	0.02	63/89	46/52	1.17(0.69‐1.97)	0.56
P53																
Negative	28/40	22/86	0.37(0.19‐0.73)	0.004	26/80	30/60	1.62(0.86‐3.06)	0.14	35/92	22/46	1.28(0.37‐2.43)	0.45	35/89	21/52	1.01(0.53‐1.92)	0.97
Positive	41/40	20/86	0.23(0.12‐0.44)	<0.001	31/80	41/60	1.73(0.97‐3.11)	0.07	30/92	40/46	2.95(1.62‐5.49)	<0.001	35/89	36/52	1.59(0.88‐2.88)	0.12

Bold font: *p* < 0.05. ER: estrogen receptor.

HER2, human epidermal growth factor receptor‐2; PR, progesterone receptor.

### 3.4 Expression quantitative trait loci

**FIGURE 1:**
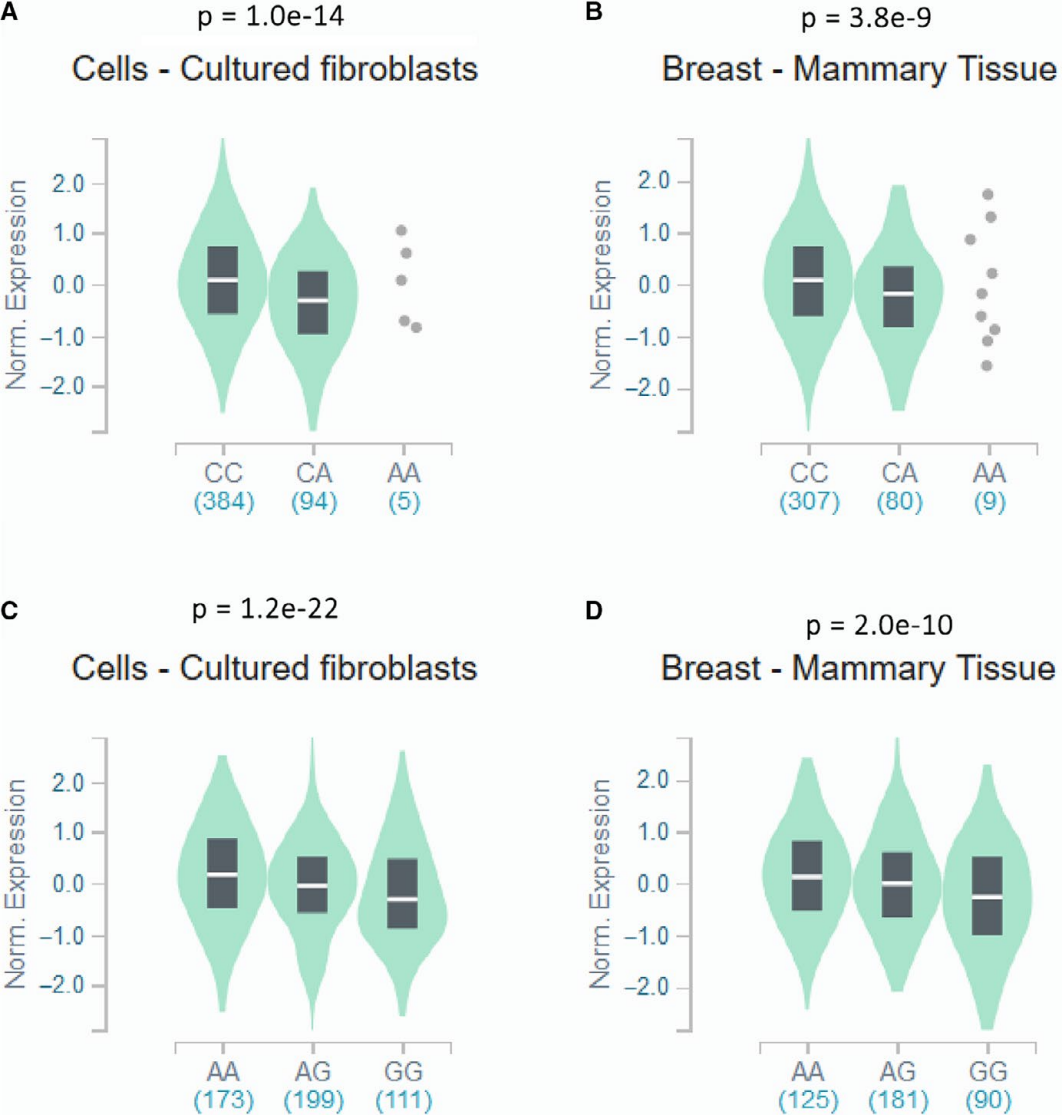
Functional implications of *ERCC1* rs2298881 and rs11615 polymorphisms. Effect of *ERCC1* rs2298881 on mRNA expression in (A) breast mammary tissues and (B) cell‐cultured fibroblasts. Effect of *ERCC1* rs11615 on mRNA expression in (C) breast mammary tissues and (D) cell‐cultured fibroblasts. The data were obtained from the GTEx (https://www.gtexportal.org/)

As shown in Figure 1, the GTEx portal was used to assess the effects of rs2298881 C>A and rs11615 G>A on *ERCC1* gene expression. We found that both rs2298881 C> A and rs11615 G>A genotypes were significantly related to *ERCC1* gene expression in breast‐mammary and tissue‐ and cell‐cultured fibroblasts.

## 4 DISCUSSION

We performed the first case–control study of the role of *ERCC1*/*XPF* polymorphisms in Han and Uygur patients with breast cancer. In particular, we included 140 Uygur patients with breast cancer, 141 Uygur healthy controls, 265 Han patients with breast cancer, and 374 Han healthy controls. Our data showed that rs2298881 C>A was associated with a higher breast cancer risk, and rs3212986 C>A and rs11615 G>A were associated with a lower breast cancer risk in the Uygur population. In addition, the rs11615 and rs2276466 polymorphisms frequencies were higher in the Uygur group than the Han group, while the opposite trend was observed for rs2298881.

*ERCC1* is located on chromosome 19q13.32 and contains 10 exons. *XPF* maps to chromosome 16p13.12 and consists of 11 exons. The proteins *ERCC1* and *XPF* act as structure‐specific endonucleases in the form of heterodimers.^18^ The heterodimer catalyzes the formation of a 5′ incision in the process of nucleotide excision and repair.^19^ In the heterodimer, *ERCC1* is a key DNA‐binding subunit without endonuclease activity, while *XPF* has catalytic activity.^20^ Associations between genetic variation in *ERCC1*/*XPF* and several human genetic diseases have been shown in previous research.^21^ Previous studies have also reported a relationship between *ERCC1*/*XPF* gene polymorphisms and cancer risk. For example, individuals with rs11615 polymorphisms are predisposed to colorectal cancer.^22^

However, in another case–control study in the United States, no association was observed between *ERCC1*/*XPF* polymorphisms and endometrial cancer susceptibility.^23^ The inconsistencies among studies indicate that the same genetic polymorphism may have different effects on susceptibility depending on race or cancer type. Therefore, it is necessary to explore the contribution of *ERCC1*/*XPF* gene polymorphisms to breast cancer risk in specific populations, including the Xinjiang Uygur and Han groups.

This is the first study of the association between *ERCC1*/*XPF* polymorphisms and susceptibility to breast cancer in Uygur and Han populations in Xinjiang. We observed that rs2298881 C>A was related to a reduced breast cancer risk, and rs3212986 C>A and rs11615 G>A were related to an increased breast cancer risk among Uygur individuals. These results were consistent with those of previous studies.^24^, ^25^, ^26^, ^27^ The opposite pattern observed for rs11615 G>A and rs2298881 C>A with respect to breast cancer susceptibility may be explained by eQTL results. The rs2298881 variant led to a decrease in *ERCC1* expression, while the rs11615 variant led to an increase in *ERCC1* expression. Among Han individuals, we failed to detect a statistically significant difference in five genetic models, contrary to the results of a previous study.^28^ This difference may be due to the different origins of the study population. Our Han group was from Xinjiang, whereas the previous study included individuals from Henan Province. This suggests that genetic polymorphisms within the same ethnic group in different regions have different effects on cancer susceptibility. Extensive evidence suggests that a single SNP may not have sufficient capacity to explain the overall cancer risk, and a combination of multiple SNPs may be a more useful predictor.^29^ Therefore, we further analyzed the combined effect of risk genotypes for *ERCC1*. We found that Uygur patients with breast cancer with 2‐3 combined risk genotypes of *ERCC1* had a higher risk. Similar conclusions have been reported for other cancers.^30^, ^31^

However, our study had some limitations. First, as a single‐center study, selection bias is inevitable. Second, the size of the Uygur group was relatively small compared to that of the Han group. Thus, our conclusions, especially those for the Uygur population, need to be verified using a larger sample size. Third, the number of SNPs analyzed in this study was limited, and it is necessary to evaluate links between additional SNPs and breast cancer susceptibility. Finally, our conclusions should be interpreted with caution because the population was from Xinjiang and generalizability to other populations has not been established.

## 5 CONCLUSIONS

In summary, our study showed that *ERCC1*/*XPF* gene polymorphisms in the Uygur group predispose individuals to breast cancer. This finding should be verified in a larger sample, and further studies are needed to determine the mechanism by which *ERCC1*/*XPF* influence breast cancer susceptibility as well as the causes of differences among races. Finally, our research deepens our understanding of the role of genetic variation in different races in cancer and may contribute to future research focused on cancer occurrence and prevention.

## CONFLICTS OF INTEREST

The authors report no conflicts of interest.

## AUTHOR CONTRIBUTIONS

H.‐T. L. and L.‐H. Z. performed experiments, analyzed data and wrote the paper; performed some experiments and analyzed data; B.‐L. M. and Z.‐J. D. initiated the study, designed experiments. J. M., Y.‐Y. Z., J.‐J. F., N. L., Y. Z., T. S., and Z. Z. read and approved the final manuscript.

## References

[1] Bray F , Ferlay J , Soerjomataram I , Siegel RL , Torre LA , Jemal A . Global cancer statistics 2018: GLOBOCAN estimates of incidence and mortality worldwide for 36 cancers in 185 countries. CA Cancer J Clin. 2018;68:394‐424. 30207593 10.3322/caac.21492

[2] Chen W , Zheng R , Baade PD , et al. Cancer statistics in China, 2015. Cancer J Clin. 2016;66(2):115‐132. 10.3322/caac.21338 26808342

[3] Fan L , Strasser‐Weippl K , Li J‐J , et al. Breast cancer in China. Lancet Oncol. 2014;15:e279‐e289. 24872111 10.1016/S1470-2045(13)70567-9

[4] Mavaddat N , Antoniou AC , Easton DF , Garcia‐Closas M . Genetic susceptibility to breast cancer. Molecular Oncol. 2010;4:174‐191. 10.1016/j.molonc.2010.04.011 PMC5527934 20542480

[5] Michailidou K , Beesley J , Lindstrom S , et al. Genome‐wide association analysis of more than 120,000 individuals identifies 15 new susceptibility loci for breast cancer. Nat Genet. 2015;47:373‐380. 25751625 10.1038/ng.3242 PMC4549775

[6] Pellegrino B , Bella M , Michiara M , et al. Triple negative status and BRCA mutations in contralateral breast cancer: a population‐based study. Acta bio‐medica: Atenei Parmensis. 2016;87:54‐63. 27163896

[7] Stadler ZK , Thom P , Robson ME , et al. Genome‐wide association studies of cancer. J Clin Oncol. 2010;28:4255‐4267. 20585100 10.1200/JCO.2009.25.7816 PMC2953976

[8] Marteijn JA , Lans H , Vermeulen W , Hoeijmakers JH . Understanding nucleotide excision repair and its roles in cancer and ageing. Nat Rev Mol Cell Biol. 2014;15:465‐481. 24954209 10.1038/nrm3822

[9] Zhu J , Fu W , Jia W , Xia H , Liu GC , He J . Association between NER Pathway Gene Polymorphisms and Wilms Tumor Risk. Molecular Ther Nucleic Acids. 2018;12:854‐860. 30161024 10.1016/j.omtn.2018.08.002 PMC6118157

[10] Zhao Z , Zhang A , Zhao Y , et al. The association of polymorphisms in nucleotide excision repair genes with ovarian cancer susceptibility. Biosci Rep. 2018;38:BSR20180114. 29669843 10.1042/BSR20180114 PMC6013708

[11] Zhuo Z‐J , Liu W , Zhang J , et al. Functional polymorphisms at ERCC1/XPF genes confer neuroblastoma risk in chinese children. EBioMedicine. 2018;30:113‐119. 29544698 10.1016/j.ebiom.2018.03.003 PMC5952228

[12] Ding JH , Huang XG , Yu B , Sun GH . The analysis of clinicopathological characteristics and prognosis in Uygurs women with breast cancer of Xinjiang. Tumor Res Clin. 2006;18:550‐552.

[13] He J , Zhuo ZJ , Zhang A , et al. Genetic variants in the nucleotide excision repair pathway genes and gastric cancer susceptibility in a southern Chinese population. Cancer Manag Res. 2018;10:765‐774. 29695933 10.2147/CMAR.S160080 PMC5903836

[14] Cheng J , Zhuo Z , Xin Y , et al. Relevance of XPD polymorphisms to neuroblastoma risk in Chinese children: a four‐center case‐control study. Aging. 2018;10:1989‐2000. 30089098 10.18632/aging.101522 PMC6128416

[15] Zhu J , Wang M , He J , et al. Polymorphisms in the AKT1 and AKT2 genes and oesophageal squamous cell carcinoma risk in an Eastern Chinese population. J Cell Mol Med. 2016;20:666‐677. 26828791 10.1111/jcmm.12750 PMC5126231

[16] Zhu J , Wang M , Zhu M , et al. Associations of PI3KR1 and mTOR polymorphisms with esophageal squamous cell carcinoma risk and gene‐environment interactions in Eastern Chinese populations. Sci Rep. 2015;5:8250. 25654238 10.1038/srep08250 PMC4318264

[17] Zhuo Z , Zhou C , Fang Y , et al. Correlation between the genetic variants of base excision repair (BER) pathway genes and neuroblastoma susceptibility in eastern Chinese children. Cancer Commun. 2020. 10.1002/cac2.12088 PMC7668499 32780923

[18] Tsodikov OV , Enzlin JH , Schärer OD , Ellenberger T . Crystal structure and DNA binding functions of ERCC1, a subunit of the DNA structure‐specific endonuclease XPF‐ERCC1. Proc Natl Acad Sci. 2005;102(32):11236‐11241 16076955 10.1073/pnas.0504341102 PMC1183572

[19] Houtsmuller AB , Rademakers S , Nigg AL , Hoogstraten D , Hoeijmakers JH , Vermeulen W . Action of DNA repair endonuclease ERCC1/XPF in living cells. Science (New York, NY). 1999;284:958‐961. 10.1126/science.284.5416.958 10320375

[20] Enzlin JH , Schärer OD . The active site of the DNA repair endonuclease XPF‐ERCC1 forms a highly conserved nuclease motif. EMBO J. 2002;21:2045‐2053. 11953324 10.1093/emboj/21.8.2045 PMC125967

[21] Niedernhofer LJ , Garinis GA , Raams A , et al. A new progeroid syndrome reveals that genotoxic stress suppresses the somatotroph axis. Nature. 2006;444:1038‐1043. 17183314 10.1038/nature05456

[22] Hou R , Liu Y , Feng YE , et al. Association of single nucleotide polymorphisms of ERCC1 and XPF with colorectal cancer risk and interaction with tobacco use. Gene. 2014;548:1‐5. 24861646 10.1016/j.gene.2014.05.025

[23] Doherty JA , Weiss NS , Fish S , et al. Polymorphisms in nucleotide excision repair genes and endometrial cancer risk. Cancer Epidemiol, Biomarkers Prevention. 2011;20:1873‐1882. 10.1158/1055-9965.EPI-11-0119 PMC3169742 21750170

[24] Zhang L , Wang J , Xu L , et al. Nucleotide excision repair gene ERCC1 polymorphisms contribute to cancer susceptibility: a meta‐analysis. Mutagenesis. 2012;27:67‐76. 22002622 10.1093/mutage/ger062

[25] He J , Xu YU , Qiu L‐X , et al. Polymorphisms in ERCC1 and XPF genes and risk of gastric cancer in an eastern Chinese population. PLoS One. 2012;7:e49308. 23166636 10.1371/journal.pone.0049308 PMC3499547

[26] He MG , Zheng K , Tan D , Wang ZX . Association between ERCC1 and ERCC2 gene polymorphisms and susceptibility to pancreatic cancer. Genetics Molecular Res. 2016;15:gmr7879. 10.4238/gmr.15017879 27051038

[27] Zhang Q , Zheng X , Li X , et al. The polymorphisms of miRNA‐binding site in MLH3 and ERCC1 were linked to the risk of colorectal cancer in a case‐control study. Cancer Med. 2018;7:1264‐1274. 29516665 10.1002/cam4.1319 PMC5911615

[28] Yang Z , Fang X , Pei X , Li H . Polymorphisms in the ERCC1 and XPF genes and risk of breast cancer in a Chinese population. Genetic Testing Molecular Biomarkers. 2013;17:700‐706. 23909490 10.1089/gtmb.2013.0122 PMC3761393

[29] Pan J , Lin J , Izzo JG , et al. Genetic susceptibility to esophageal cancer: the role of the nucleotide excision repair pathway. Carcinogenesis. 2009;30:785‐792. 19270000 10.1093/carcin/bgp058 PMC2675653

[30] Tse D , Zhai R , Zhou W , et al. Polymorphisms of the NER pathway genes, ERCC1 and XPD are associated with esophageal adenocarcinoma risk. Cancer Causes Control. 2008;19:1077‐1083. 18478337 10.1007/s10552-008-9171-4 PMC3106102

[31] He J , Wang F , Zhu J , et al. Association of potentially functional variants in the XPG gene with neuroblastoma risk in a Chinese population. J Cell Mol Med. 2016;20:1481‐1490. 27019310 10.1111/jcmm.12836 PMC4956948

